# Preparation and Characterization of Vitamin C Aquasomes

**DOI:** 10.2174/0126673878352032250325002719

**Published:** 2025-04-08

**Authors:** Fernando Bwalya, Liban Barre, Murat Erdem, Mustafa Sinan Kaynak

**Affiliations:** 1 Department of Pharmaceutical Technology, Graduate School, Anadolu University, Eskisehir, Turkey;; 2 Department of Chemistry, Faculty of Science, Eskişehir Technical University (ESTU), Eskisehir, Turkey;; 3 Department of Pharmaceutical Technology, Faculty of Pharmacy, Anadolu University, Eskisehir, Turkey

**Keywords:** Hydroxyapatite, vitamin C, ceramic nanoparticles, aquasomes, water-like bodies, colloidal precipitation, drug delivery

## Abstract

**Introduction:**

Aquasomes are water-based nanocarriers widely used in pharmaceutical applications for the delivery of various molecules.

**Aim:**

This research examines the preparation and characterization of vitamin C-loaded aquasomes.

**Methods:**

The aquasomes were prepared by using colloidal precipitation and sonication methods. Various characterizations, including particle size, polydispersity index (PDI), and zeta potential, were performed on the core, lactose coating, and final formulation of vitamin C-loaded aquasomes. Further analysis was carried out using Scanning Electron Microscopy-Energy Dispersive X-Ray (SEM-EDX), X-ray Diffraction (XRD), Fourier Transform Infrared Spectroscopy (FTIR), Thermogravimetric Analysis (TGA), and Differential Scanning Calorimetry (DSC). Additionally, the *in vitro* release profile of vitamin C from the aquasomes was compared with that of a commercially available vitamin C formulation and a shelf-life analysis was conducted.

**Results:**

The addition of lactose and vitamin C led to an increase in the particle size of the core, from 348 nm to 654 nm, while the zeta potential decreased from -31.9 mV to -12.8 mV. The percent payload was found to be 52.63%. TGA results indicated weight loss in HAP, suggesting thermal degradation, while DSC analysis revealed the melting points of lactose sugars and the thermal behavior of vitamin C. The dissolution results show that vitamin C-loaded aquasomes released 4-6% more of the vitamin in acidic (pH 1.2) and phosphate buffer (pH 6.8) environments over 90 minutes, compared to commercial vitamin C products. The aquasomes exhibited excellent stability, maintaining over 90% of their potency over 90 days.

**Conclusion:**

Vitamin C-loaded aquasomes have been successfully prepared and demonstrated better performance compared to commercial products. This study suggests that aquasomes keep vitamin C stable and may improve its absorption in the body.

## 1 INTRODUCTION

Vitamin C, also known as ascorbic acid, is a water-soluble vitamin known for its potent antioxidant properties within the human body [1, 2]. Historically recognized for its role in preventing scurvy among sailors and pirates at sea, vitamin C has since been acknowledged for its ability to boost white blood cell production, combat free radicals, promote collagen synthesis crucial for skin and hair health, and safeguard the skin from UV radiation and damage [3-5].

The recommended daily intake is 75 mg for women and 90 mg for men. Rich sources of vitamin C include vegetables like broccoli, Brussels sprouts, tomatoes, and bell peppers, along with citrus fruits and a variety of berries [6, 7].

For individuals who find it challenging to meet their vitamin C needs through food alone, supplements offer a convenient alternative. Additionally, when taken with iron supplements, vitamin C can enhance iron absorption. Its combination with oil-soluble vitamin E further strengthens the body’s defense against free radicals [8].

Vitamin C, while essential for health, poses challenges in formulation and delivery due to its instability [9]. Ascorbic acid is highly sensitive to factors like light, heat, and oxygen which can cause it to break down quickly and lose effectiveness in pharmaceutical and nutraceutical products [10]. This instability makes it difficult to include vitamin C in foods, supplements, or other delivery methods where maintaining its potency is crucial [11].

The use of aquasomes for vitamin C delivery offers several potential advantages. Aquasomes can aid in preserving vitamin C by serving as a barrier against light, heat, and oxidation [12]. Aquasomes have also shown promise in enhancing the delivery and stability of vitamin C. Known for their nano-size, these drug carriers have been proven to boost the therapeutic effectiveness of medicinal substances by controlling their release, improving stability, and prolonging circulation time by safeguarding the medication from phagocytosis and premature degradation [13-15]. Aquasomes are three-layered self-assembled structures. They consist of a core material, such as HAP, calcium phosphate, or ceramic, coated with disaccharides like lactose, cellobiose, or trehalose, onto which the active pharmaceutical ingredient (API) is adsorbed [16-18]. Aquasomes are sometimes described as “bodies of water ” and they are capable of preserving various therapeutic molecules [19]. This distinctive design is well suited for enclosing drugs such as vitamin C to ensure their stability during transport and storage. In this study, lactose was used to coat the core, contributing to the creation of a hydrophilic environment for the aquasomes. As a polyhydroxy oligomer, lactose enhances the surface coating's ability to stabilize labile bioactive agents, such as vitamin C, by preventing dehydration. This is achieved through the formation of a quasi-aqueous environment and the adsorption of vitamin C onto the surface of the coating. Fig. (**1**) illustrates the structures of HAP, lactose, and vitamin C [20].

Aquasomes are composed of a biodegradable ceramic core with negligible hemolytic activity [21, 22]. They have been explored for delivering a variety of bioactive molecules, including drugs, proteins, enzymes, and vaccines. They are used in the delivery of anticancer drugs like etoposide [23], glucose-regulating insulin [24], anti-inflammatory agents such as indomethacin and lornoxicam, enzymes like serratiopeptidase [12, 25] vaccines such as hepatitis B [26] as well as a carrier for hemoglobin [27]. The purpose of this study is to prepare and characterize vitamin C-loaded aquasomes. Furthermore, the aquasomes were investigated for dissolution profile in acidic (pH 1.2) and phosphate buffer (pH 6.8). Lastly, the stability of the vitamin C aquasomes over 3 months was investigated.

## 2 MATERIALS AND METHODS

### 2.1 Materials

Calcium chloride and methanol were procured from Merck-Germany, monobasic sodium phosphate from Carlo Erba Reagents-Germany, lactose from Drogsan-Turkey, hydrochloric acid from Sigma Aldrich-USA, vitamin C powder was procured from Koç İlaç-Turkey, a 0.22 μm filter paper, and phosphate buffer, and distilled water were obtained from Anadolu University-BIBAM-Turkey. All materials were of analytical grade.

### 2.2 Preparation of Nanohydroxyapatite

8.9 g of disodium hydrogen phosphate and 7.35 g of calcium chloride were dissolved separately in 60 ml of distilled water each. The disodium phosphate solution was added dropwise to the calcium chloride solution with continuous agitation [28]. The chemical reaction (equation 1) is represented by:

2Na_2_HPO_4_+3CaCl_2_+H_2_O→Ca_3_[PO_4_]_2_+4NaCl+2H_2_+Cl_2_+ [O] (1)

Following this, both solutions were sonicated (Sonicator, WUC-A03H Model, Daihan Scientific Company Limited-Korea) at 50 W for 2 hours at 4°C. The calcium phosphate core was then separated by centrifugation (Nüve-Turkey), washed with distilled water, filtered, and dried at 50°C in a hot air oven. Calcium phosphate was further calcined at 1000°C in a Malvern furnace, resulting in the formation of a hydroxyapatite core, which was then finely ground using a mortar and pestle [29, 30].

### 2.3 Coating of the Core

100 mg of inorganic cores were resuspended in distilled water and mixed with a lactose solution (5 mg/mL). After agitation and incubation, the solution underwent centrifugation, filtration, and lyophilization (Freeze Dryer, Operon FDB-8602 Model -Korea) to obtain a polyhydroxylated core [31].

### 2.4 Preparation of Vitamin C-loaded Aquasomes

Vitamin C solution (5 mg/mL) was combined with the sugar-coated core in a 1:1 ratio and subjected to vigorous shaking for 1 hour. After centrifugation, the drug-loaded aquasomes were lyophilized [17].

### 2.5 Characterization of Aquasomes

Various physicochemical and morphological characterizations were conducted on HAP, lactose-coated HAP, and vitamin C-loaded aquasomes. The particle size, PDI, and zeta potential of the aquasomes were determined using a ZetaSizer (Malvern Instruments Ltd., Worcestershire, UK). The samples were diluted with distilled water and then transferred into a cuvette, and the analysis was performed. All measurements were conducted at room temperature with three replicates [32]. SEM-EDX, XRD, and FTIR analyses were also performed. For SEM analysis, the prepared aquasomes samples were completely dry, free of moisture or residues, and in solid form capable of withstanding vacuum conditions. They were then mounted on aluminum stubs using double-sided carbon tape and coated with a thin conductive layer of carbon using the SPC-900 sputtering device to enhance conductivity and prevent charging. The analysis was performed using the Hitachi TM3030 Plus Tabletop Scanning Electron Microscope (Hitachi, Japan), with images captured at different magnifications for high-resolution imaging. Morphological and elemental composition analyses of the samples were successfully obtained [33]. In the case of XRD analysis, the samples were finely ground using mortar and pestle to ensure a uniform powder. The powdered samples were mounted on glass slides, and analysis was performed using a Rigaku MiniFlex 600 diffractometer. Data were collected at a scanning rate of 2° per minute over a 2θ range of 0° to 55° [34]. FTIR spectra were obtained using a PerkinElmer Spectrum 100 spectrometer. The samples were prepared using the KBr pellet method, where the dried sample was mixed with potassium bromide (KBr) and pressed into a pellet. Spectra were recorded in the range of 4000-600 cm^-1^ [35].

Thermogravimetric analysis (TGA) and DSC evaluation were also performed. TGA involves tracking weight changes relative to temperature within a regulated environment, following a predefined heating schedule. On the other hand, Differential Scanning Calorimetry (DSC) measures the variance in heat flow into or out of a material compared to a standard reference under controlled heating or cooling conditions [36]. TGA was utilized to determine weight loss percentages in Hydroxyapatite (HAP), lactose-loaded HAP, and vitamin C-loaded aquasomes. This analysis used a TA-60WS thermal analyzer (TA Instruments-SDT 650-USA). The process involved heating samples in alumina pans at 10°C per minute, with a nitrogen flow environment, across a temperature spectrum of 30 to 800°C. Differential scanning calorimetry (DSC) was employed to verify the multilayer coating of the aquasomes using a DSC (TA Instruments-SDT 650-USA).

### 2.6 *In-vitro* Drug Release Studies

Dissolution studies were performed as per USP/NF standards using the Pharmatest Apparatus, Germany. Capsules containing vitamin C-loaded aquasomes were analyzed, and samples were taken at predetermined intervals. The aliquots were filtered, properly shaken using a vortex, and transferred to a volumetric flask. The solution's absorbance was measured, and a calibration curve was plotted. The absorbance of the vitamin C was λ_max_ 245 nm using a UV Visible Spectrophotometer (Shimadzu, Japan). The cumulative % release was calculated and plotted as a function of time. The data were analyzed to assess the statistical significance between commercial vitamin C products and vitamin C-loaded aquasomes using t-tests in Microsoft Excel 2019, with a *p*-value <0.05 considered statistically significant.

### 2.7 Payload

The payload was calculated according to the formula below:

% Payload = (Amount of drug in aquasomes / Amount of aquasomes) × 100.

### 2.8 Shelf-life Assessment of Vitamin C

Capsules were stored at room temperature and analyzed for vitamin C content at specific intervals, with all measurements performed in triplicate.

## 3 RESULTS AND DISCUSSION

### 3.1 Particle Size

The HAP image shows a granular and irregular structure. The particles seem to be agglomerated with some level of porosity. This kind of structure is usual for HAP and implies a crystalline structure. When coated with lactose the shape appears to alter. The particles look smoother indicating that lactose has formed a coating around the HAP particles. Lactose fills the gaps or spaces between the HAP particles. Additionally, the adsorption of vitamin C showed a dense and agglomerated appearance.

Vitamin C-loaded aquasomes were synthesized by first preparing an inorganic core (of Hydroxyapatite) that was subsequently coated with lactose. The drug, vitamin C, was then adsorbed onto this lactose-coated core. In existing literature, the focus on vitamin C-loaded aquasomes is limited. Prior research formed lactose-coated aquasomes using Indomethacin, Piroxicam, [37] and Lornoxicam. Their method involved sonication and structural characterization using X-ray powder diffractometry, transmission electron microscopy, and scanning electron microscopy. The Indomethacin-loaded aquasomes were spherical and exhibited a smaller size (averaging between 60–120 nm) [38] compared to our vitamin C aquasomes, which measured at 654 nm, as shown in Fig. (**2**) and Table **1**. HAP was chosen as the core material for aquasomes due to its biocompatibility, bioactivity, and structural similarity to human bone. Its tunable surface properties, high porosity, and stability make it ideal for efficient drug adsorption and delivery, ensuring the protection and stabilization of labile molecules like vitamin C [39]. The decrease in zeta potential after coating with lactose and adsorption of vitamin C can be attributed to the presence of -OH and COO- groups in their structure. These groups interact with the particle surface, modifying the surface charge and altering the overall zeta potential, which can influence the stability of the aquasomes [40]. Energy Dispersive X-ray (EDX) analysis, integrated with scanning electron microscopy, is a versatile technique for elemental identification and quantification, widely applied in various fields including drug delivery studies. The SEM-EDX analysis of the HAP core, lactose-coated HAP, and vitamin C aquasome formulations aligned with their respective elemental compositions, as shown in Fig. (**3**). The coating process showed a reduction in calcium levels accompanied by an increase in oxygen, indicating the adsorption of vitamin C [41, 42].

Zeta potential (ζ-potential) is a crucial parameter that reflects the electrostatic interactions and stability of nanoparticle dispersions, influenced by conditions like pH and ionic strength. Nanoparticles with zeta potential values ranging from ± 30 to ± 40 mV are regarded as moderately stable, whereas values greater than ± 40 mV signify robust electrostatic stability. It serves as a reliable measure for evaluating the colloidal stability of nanoparticles [43]. In our study, the zeta potential decreased with the addition of lactose and further with vitamin C, suggesting changes in surface charge and potentially reduced colloidal stability. A decrease in zeta potential can signify reduced repulsion between particles, which might lead to more aggregation [44]. However, the observed zeta potential shows average stability. A PDI value closer to 0 indicates a more uniform particle size distribution, while a value closer to 1 suggests a wide distribution of particle sizes. The PDI increases to 0.531 with the addition of lactose and further to 0.549 with vitamin C, suggesting that the formulations become more heterogeneous in size with each addition. The average particle size increases with each formulation step, starting from 348 nm for the hydroxyapatite core, to 454 nm with lactose, and significantly to 654 nm with the addition of vitamin C. This trend suggests that adding lactose and vitamin C contributes to the growth of the particles.

### 3.2 FTIR Analysis

Based on the characteristic bands observed in Fig. (**4**), HAP, lactose, and vitamin C can be confirmed in the final formulation.

**Fig. (4):**
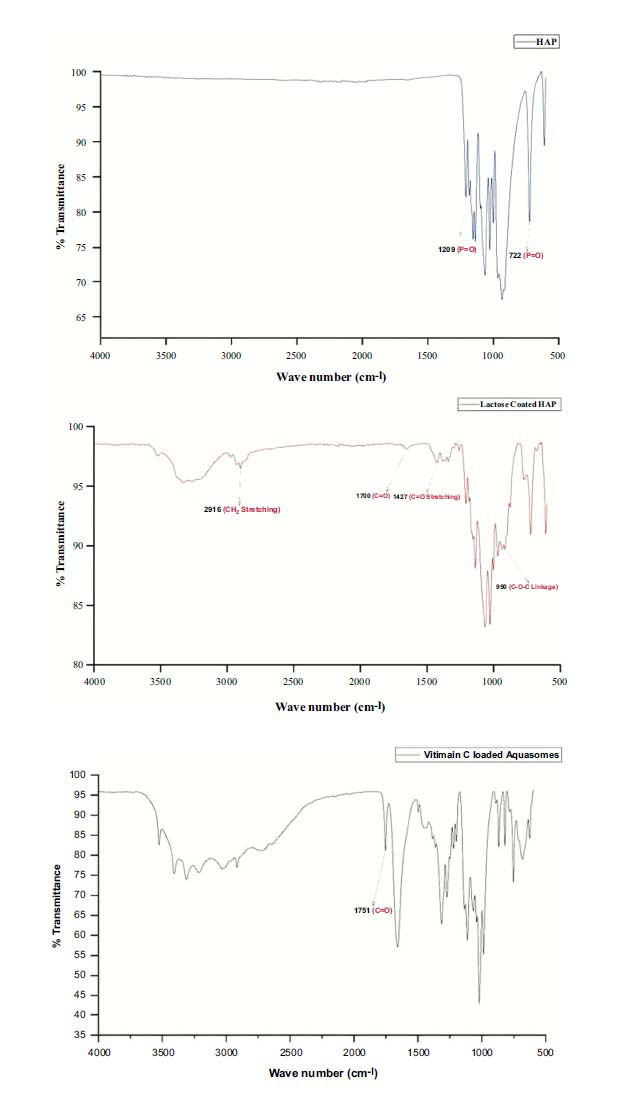
FTIR Spectra of HAP, lactose-Coated HAP, and vitamin C-Loaded AQ.

FTIR spectroscopy (Fig. **4**) provides evidence of the sugar coating on the core. The FTIR spectra bands correspond to the functional groups intrinsic to both the core and the lactose coating, as detailed in Table **2** [45-49]. Additionally, the FTIR analysis confirmed the presence of vitamin C within the aquasomes, further validating the successful adsorption of the drug onto the lactose-coated ceramic core. Characteristic bands corresponding to the PO_4_^3−^ groups in HAP were evident at wavenumbers 473, 555, 611, 723, and 1328 cm^−1^ [34, 50]. The 3600 and 3200 cm^-1^ bands signify hydroxyl groups engaged in intermolecular hydrogen bonding. Notably, the bands near 3000 cm^-1^ are representative of C-H bond stretching, situated within the 3000 to 2400 cm^-1^ region.

Furthermore, specific bands in 1764 and 1675 cm^-1^ are associated with axial deformation vibrations of C=O in the γ-lactone ring and the double bond adjacent to the -O- group, respectively. The activation of γ-lactone molecules instigates an enhanced carbonyl uptake and, due to the proximity of the double bond, leads to pronounced absorption within the 1685 to 1660 cm^-1^ range for the C=C group. Between 1500 and 1200 cm^-1^, several absorption bands characteristic of angular strain vibrations of C-H in CH_2_ and CH groups. The bands within the 1277 to 1046 cm^-1^ spectrum indicate axial deformation C-O of alcohols, and the angular deformation of O-H is discernible in the 990 to 1027 cm^-1^ region [36].

### 3.3 XRD Analysis

X-ray powder diffraction analysis of all samples allowed us to identify Hydroxyapatite's characteristic hexagonal and triclinic crystalline system at 2θ, as shown in Fig. (**5**).

The crystalline nature of HAP was confirmed using X-ray diffraction (XRD) analysis, as depicted in Fig. (**5**). The observed XRD pattern, showcasing pronounced peaks between 25–35° (2θ angle), affirms the crystalline behavior of HAP. This pattern closely aligns with the standard HAP diffractogram the International Centre for Diffraction Data (ICDD) provided (JCPDS reference no. 9-432) [51, 52]. No new peaks appeared after coating, as lactose and vitamin C are not crystals, and the basic crystal structure of HAP was maintained throughout [34, 38].

### 3.4 TGA and DSC Analysis

The thermal stability of the formulations was characterized using TGA, and the weight loss patterns of HAP, lactose-coated HAP, and vitamin C-loaded AQ are shown in Fig. (**6a-c**). In Fig. (**6a**) the TGA curve (green) indicates weight loss suggesting that HAP remains stable at high temperatures. The DSC curve (blue) does not exhibit exothermic reactions implying no major phase transitions or degradation events within the measured temperature range. In Fig. (**6b** and **6c**) the TGA curves show weight reduction due to lactose and vitamin C breakdown as temperature increases. Additionally, the DSC curves display multiple peaks indicating processes like melting or decomposition for lactose and a series of endothermic and exothermic changes for vitamin C upon heating. At 563°C in Fig. (**6b**) the TGA curve reveals that 19.17% of lactose remains losing than 80% of its mass with rising temperature. Similarly, at 510.54°C in Fig. (**6c**) 9.437% of the weight of vitamin C-loaded Aquasome is retained according to the TGA curve data indicating a loss of over 90% of its mass.

The HAP core showed only a slight weight decrease of 0.5%. After heating for dehydroxylation, it regains the hydroxyl groups when exposed to moisture at temperatures below 500°C [53]. Therefore, this minimal weight loss is likely due to the release of water that was both physically absorbed and chemically bonded within the structure. In contrast, a study by Damera *et al.*, indicated a 4% reduction in weight [40] while another study observed a 2.3% mass loss in synthetic hydroxyapatite [54]. The weight loss between 200 and 600°C is linked to the denaturation in lactose and vitamin C [1]. The DSC graphs show an exothermic peak around 350°C indicating the crystallization of HAP [55]. The endothermic peaks observed at 151°C and 218°C for lactose may be related to the melting points of its sugars—glucose and galactose. Glucose melts at around 146°C, galactose at 167°C, and lactose, at about 203°C [56]. Moreover, exothermic peaks at 537°C and a significant one at 400°C reveal the decomposition of lactose coated on HAP. Regarding vitamin C-loaded aquasomes, the endothermic peaks at 131°C and 170°C show when vitamin C starts to decompose while the temperatures of 380°C and 510°C indicate the breakdown of the aquasome structure itself. Vitamin C typically melts between 190-192°C [57]. This study found that vitamin C aquasomes had a biphasic weight loss pattern, at different temperatures, which may suggest differences, in the carrier matrix when compared to what Junior *et al.* (2018) reported [36].

### 3.5 Dissolution Test Results of Vitamin C-loaded Aquasomes

**Fig. (7):**
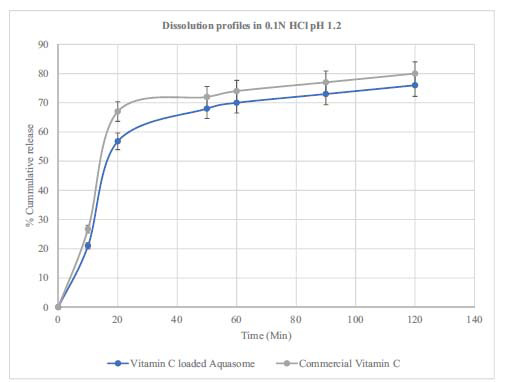
Drug release in 0.1 N hydrochloric acid solution.

**Fig. (8):**
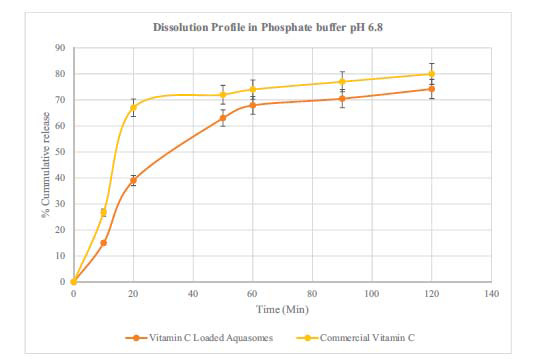
Drug release in phosphate buffer.

The dissolution profile of vitamin C-loaded aquasomes was compared to a commercial vitamin C product. The findings showed that the amount of vitamin C released in hydrochloric acid (HCl) increased rapidly at the start reaching 56% in the 20 minutes before plateauing. On the other hand the release, in phosphate buffer increased more slowly. The pH level significantly influences the release rate of vitamin C from aquasomes. The graph for the commercial vitamin C showed release rates reaching 80% after 120 minutes. The paired t-test results indicate a significant difference in the dissolution profile between vitamin C-loaded aquasomes and commercial vitamin C. In 0.1 N HCl, the t-statistic was 3.98 with a *p*-value of 0.0073, and in phosphate buffer, the t-statistic was 2.86 with a *p*-value of 0.0288. These findings suggest that the formulations dissolve differently in these media. The release profile (Figs. **7** and **8**) of vitamin C from aquasomes shows a controlled and slightly extended pattern compared to commercial vitamin C [58]. Even though the difference is small (4-6%), it is statistically significant suggesting that aquasomes can effectively regulate how vitamin C is released. This release along with the stability provided by the core of aquasomes could be advantageous in maintaining steady levels of vitamin C in the body, especially in acidic environments where the vitamin breaks down quickly [12, 19, 24, 59-61]. Future studies should focus on *in vivo* testing and exploring the use of aquasomes for delivering other vitamins or bioactive substances to fully understand their advantages as a drug delivery system.

### 3.6 Payload

The percent payload of the vitamin C aquasomes was measured to be 52.63%. Aquasomes have a large surface area compared to their size allowing them to transport more vitamin C effectively. This is due to their surface volume which may facilitate vitamin absorption.

### 3.7 Shelf-Life Assessment

**Fig. (9):**
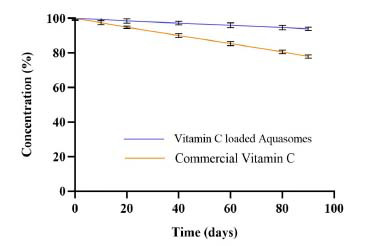
Vitamin C shelf-life assessment at room temperature during 90 days of storage.

The concentration of ascorbic acid was measured at various time points: 0, 10, 20, 40, 60, 80, and 90 days, at room temperature 25°C. The graph shows a 9% reduction in vitamin C-loaded Aquasome concentration and a 22% reduction for the commercial product after 90 days (Fig. **9**).

Aquasomes offer a protective environment that moderately stabilizes vitamin C (sensitive to various external factors, including light, heat, moisture, and oxygen), slowing its degradation rate. This is more stable than chitosan nanoparticles [62, 63]. While still retaining a significant portion of its initial concentration, the commercial vitamin C showed a more consistent decline. This could be due to the absence of a protective carrier system, making the ascorbic acid more susceptible to degradation through exposure to the various factors that affect its stability [57]. The nanosize of aquasomes with their colloidal properties allows them to bypass reticuloendothelial system clearance and concentrate effectively in the liver and muscles, where vitamin C exerts its action [64]. The ceramic core degrades through the action of monocytes, osteoclasts, and other multicellular cells, making aquasomes a promising platform for targeted and controlled drug delivery. A recent study developed berberine-loaded aquasomes with a particle size of around 263.57 nm and a zeta potential of approximately −21.0 mV to improve skin permeability for psoriasis treatment. The formulation demonstrated high drug adsorption efficiency and controlled release, with *in-vivo* studies showing a significant reduction in psoriasis symptoms and inflammatory cytokines. This research emphasizes the potential and versatility of aquasomes as an effective nanocarrier for various routes and the possibility of loading diverse bioactive [65]. The developed vitamin C aquasomes exhibited properties similar to those reported in the literature. While physicochemical and morphological characteristics may vary depending on the core material and preparation methods, the formulation remained consistent with standard aquasome properties, further confirming the success of aquasomes formulation. This study explored the potential of aquasome nanoparticles as an effective carrier for vitamin C, highlighting their promise in improving the delivery and stability of this vital nutrient.

## CONCLUSION

In this study, we explored the potential of aquasomes, a nanoparticulate carrier system, for the effective delivery of vitamin C. Hydroxyapatite, an inorganic compound, served as the core, which was subsequently coated with lactose, facilitating the adsorption of vitamin C onto it. Electron microscopy and particle size analysis showed the nanometric size of the HAP (348 nm), lactose-coated HAP (454 nm), and the vitamin C-loaded aquasomes (654 nm). The zeta potential measurements were found to be -31.9 Mv for HAP, -17.6 mV for lactose-coated HAP, and -12.8 mV for the vitamin C loaded. The size of the aquasomes, as underscored by various studies, has profound implications for their efficacy as drug delivery systems, enhancing solubility and bioavailability. The percent payload was found to be 52.63%. The crystalline nature of HAP was further validated through XRD and FTIR spectroscopy, with results closely aligning with standard data. Thermal analysis confirmed the presence of multilayer coatings on HAP. It indicated distinct thermal events for lactose and vitamin C, with implications for their thermal stability and integrity within the aquasomes. Furthermore, the dissolution medium plays a critical role in the release kinetics of vitamin C from the aquasomes. The vitamin C-loaded aquasomes would provide a slightly more prolonged release and thus benefit patients' therapeutic outcomes.

## Figures and Tables

**Fig. (1):**
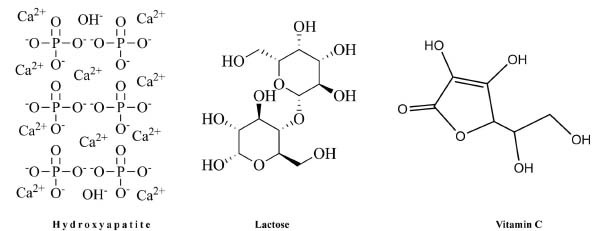
The chemical structure of hydroxyapatite, lactose and vitamin C were illustrated using ChemDraw [21].

**Fig. (2):**
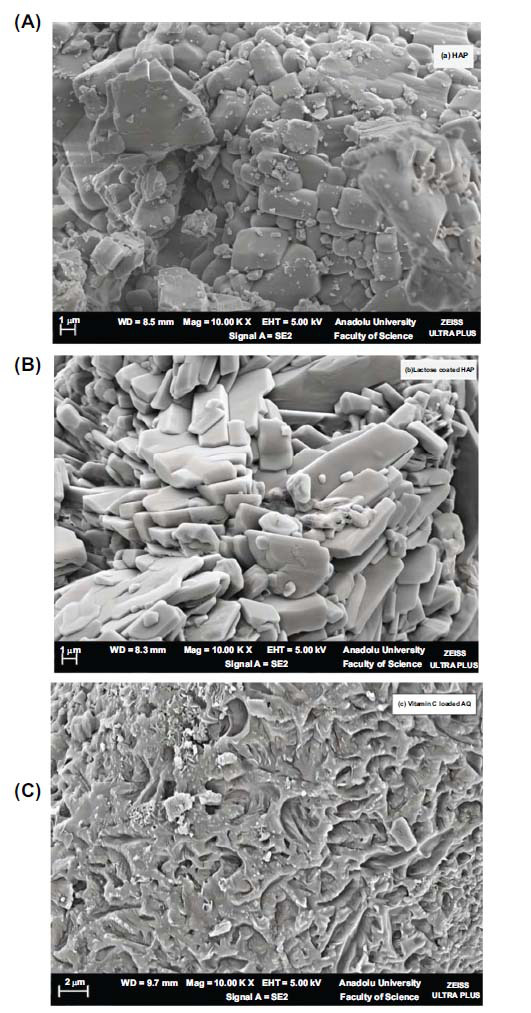
SEM images of (**a**) granular HAP core, (**b**) smoother, lactose-coated HAP, and (**c**) denser, agglomerated vitamin C-loaded AQ.

**Fig. (3):**
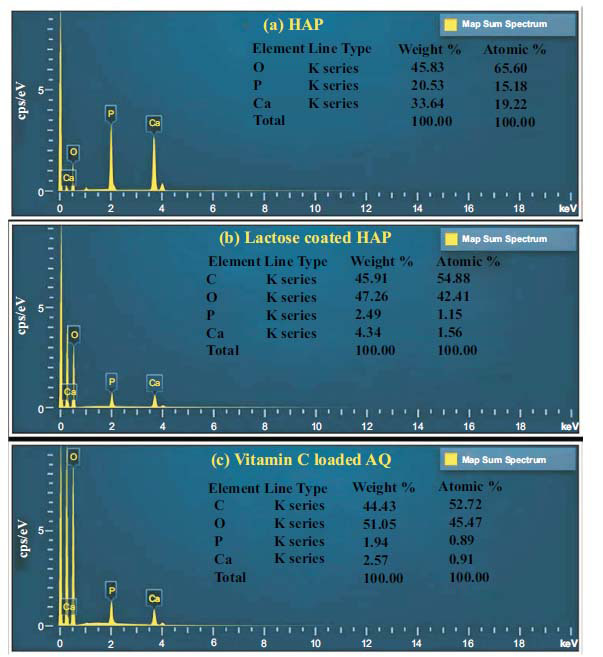
EDX spectra illustrating the stepwise formulation process: (**a**) pure hydroxyapatite (HAP) core, showing characteristic calcium, phosphorus, and oxygen signals; (**b**) lactose-coated HAP, where the appearance of carbon peaks confirms successful coating; and (**c**) final vitamin C-loaded AQ formulation, with carbon signals reflecting the incorporation of vitamin C.

**Fig. (5):**
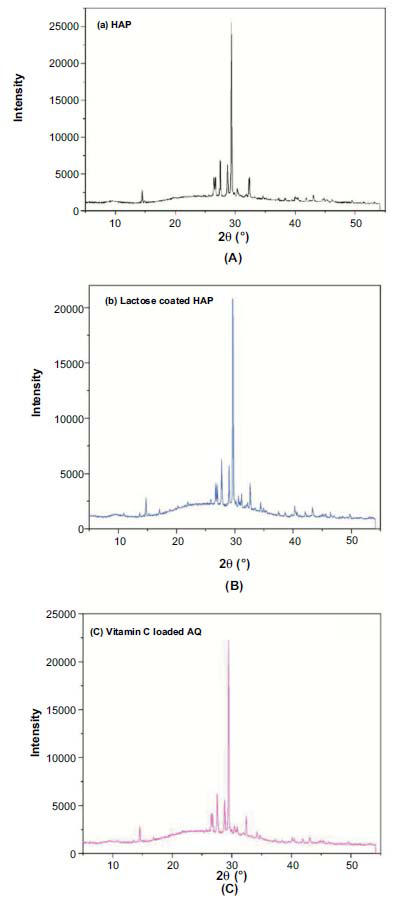
XRD patterns of HAP (**a**), Lactose coated HAP (**b**), and vitamin C loaded AQ (**c**).

**Fig. (6):**
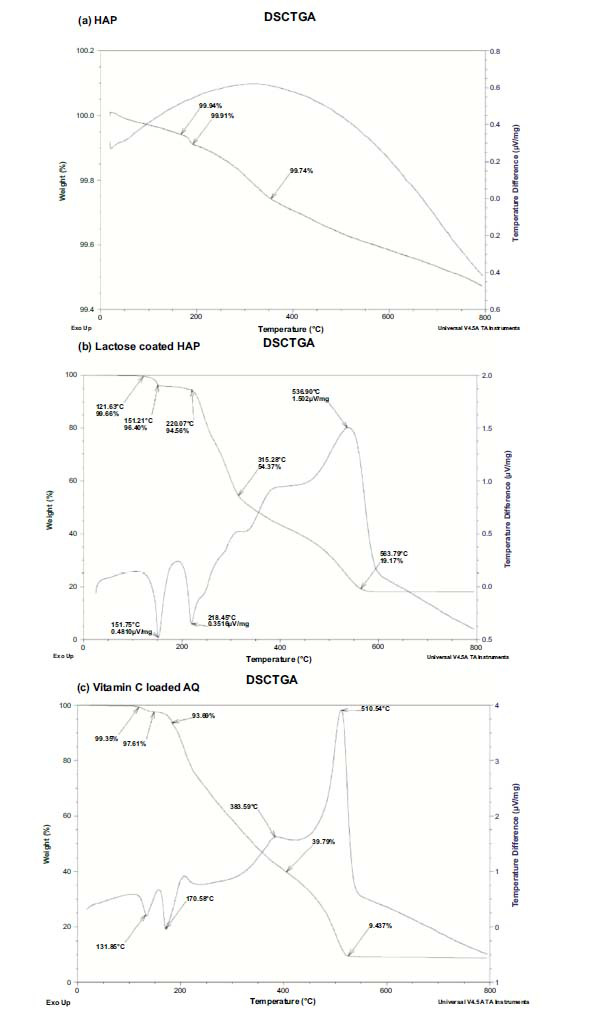
TGA and DSC results of (**a**) DSC/TGA of HAP, (**b**) Lactose-coated HAP and (**c**) Vitamin C loaded AQ.

**Table 1:** Observed particle size, PDI, and zeta potential of HAP, HAP+Lactose, HAP+Lactose+VitC.

**Formulations**	**Zeta Potential** **(mV)**	**PDI**	**Particle Size** **Average (nm)**
**HAP**	-31.9 ± 0.5	0.458 ± 0.3	348 ± 10.7
**HAP+Lactose**	-17.6 ± 0.4	0.531 ± 0.5	454 ± 16.9
**HAP+Lactose+VitC**	-12.8 ± 0.5	0.549 ± 0.4	654 ± 21.4

**Table 2:** Comparison of characteristic IR bands of the final formulation.

**Material**	**Characteristic Bands**	**Observed in this Study** **(cm^-1^)**	**Literature Values** **(cm^-1^)**	**Literature**
**Core**	Phosphate (P-O)	723	725-845	[5]
	Phosphate (P=O)	1328	1240-1300	[46]
** Lactose**	CH_2_ stretching	2916	2800-3000	[28 , 47]
	Carbonyl (C=O)	1700	1750	[19 , 28]
	Hydroxyl	-	1400-1600	-
	C-O-C Linkages	950-1300	1000-1300	[19 , 28 , 48]
	C-O Stretching	1300-1400	1000-1300	[19 , 28]
** Vitamin C**	Hydroxyl	3200-3600	3200-3600	[36 , 49]
	Carbonyl	1751	1750	[36 , 49]
	CH Stretching	2400-3000	2800-3000	[36 , 49]

## Data Availability

The authors confirm that the data supporting the findings of this research are available within the article.

## LIST OF ABBREVIATIONS

API: Active Pharmaceutical Ingredient
DSC: Differential Scanning Calorimetry
EDX: Energy Dispersive X-ray
FTIR: Fourier Transform Infrared Spectroscopy
HAP: Hydroxyapatite
ICDD: International Centre for Diffraction Data
mV: Millivolts
PDI: Polydispersity Index
pH: Potential of Hydrogen (Scale Used to Specify Acidity or Alkalinity)
SEM: Scanning Electron Microscopy
TGA: Thermogravimetric Analysis
USP/NF: United States Pharmacopeia/National Formulary
UV: Ultraviolet
XRD: X-ray Diffraction
λ_max_: Wavelength of Maximum Absorption
